# Supplementary material for: Comparison of Campylobacter jejuni Slaughterhouse and Surface-Water Isolates Indicates Better Adaptation of Slaughterhouse Isolates to the Chicken Host Environment
Source: Microorganisms. 2020 Oct 30;8(11):1693. doi: 10.3390/microorganisms8111693 (PMC7693524; doi:10.3390/microorganisms8111693)
Supplement: Supplementary file 1 [file microorganisms-08-01693-s001.pdf]

Supplement

Supplementary Table S1. Isolate designation with characteristics [1], including *flaA* nucleotide and peptide type; multilocus sequence typing (MLST) based clonal complex (CC) and sequence type (ST); mPCR clade; source and original study designation with additional data of the original microbial collection of the Biotechnical Faculty (University of Ljubljana, Slovenia).

Isolate designation	*flaA* nucleotide type	*flaA* peptide type	MLST CC	MLST ST	mPCR clade	Source	Designation in original study (Kovač et al., 2018)	Designation in collection	Year of isolation
Slaughterhouse									
S1	222	33		905	9i	Frozen chicken skin	P-KL-II ZK2	B949	2012
S2	222	33	ST-354	354	8	Water after stunning	P-KL-VOBC BB1	B955	2012
S3	222	33	ST-354	354	8	Equipment after slaughter	P-KL-VOBC BB2	B957	2012
S4	222	33	ST-354	354	8	Worker gloves after slaughter	P-KL-II ROK BB2	B964	2012
S5	34	1	ST-354	2863	1	Water	Z-A5 VB	B972	2012
S6	34	1	ST-354	2863	5	Chicken skin before cooling	Z-KL-II KC7	B975	2012
S7	34	1	ST-354	2863	5	Cecum	Z-KL-II F5	B977	2012
S8	34	1	ST-354	2863	1	Chicken skin after cooling	Z-KL-II KH1	B979	2012
Surface water									
W1	239	9	ST-45	583	9ii		266	B987	2007
W2	36	1	ST-21	104	C4/6	River water	176	B988	2007
W3	161	3	ST-22	2497	9i		258	B989	2007
W4	1285	1		5207	9ii		1603	B718	2008
W5	5	5	ST-658	1044	9ii		1604	1604	2008
W6	636	67		1367	9ii	Water	17697/26	B751	2006
W7	5	5	ST-658	657	9ii		816	B935	2008
W8	1550	46			9i		07/807	B640	2007

References

1. Kovač, J.; Stessl, B.; Čadež, N.; Gruntar, I.; Cimerman, M.; Stingl, K.; Lušicky, M.; Ocepek, M.; Wagner, M.; Smole Možina, S. Population structure and attribution of human clinical *Campylobacter jejuni* isolates from central Europe to livestock and environmental sources. *Zoonoses Publ. Health* **2018**, *65*, 51–58.
